# Albumin-Like Protein is the Major Protein Constituent of Luminal Fluid in the Human Endolymphatic Sac

**DOI:** 10.1371/journal.pone.0021656

**Published:** 2011-06-29

**Authors:** Sung Huhn Kim, Un-Kyoung Kim, Won-Sang Lee, Jinwoong Bok, Jung-Whan Song, Je Kyung Seong, Jae Young Choi

**Affiliations:** 1 Department of Otorhinolaryngology, Yonsei University College of Medicine, Seoul, Korea; 2 Department of Biology, Kyungpook National University, Daegu, Korea; 3 Department of Anatomy, Yonsei University College of Medicine, Seoul, Korea; 4 Department of Otorhinolaryngology, Ajou University College of Medicine, Suwon, Korea; 5 Laboratory of Developmental Biology and Genomics, BK21 Program for Veterinary Science, Research Institute for Veterinary Science, College of Veterinary Medicine, Interdisciplinary Program for Bioinformatics and Program for Cancer Biology, Seoul National University, Seoul, Korea; The Research Institute for Children, United States of America

## Abstract

The endolymphatic sac (ES) is an inner ear organ that is connected to the cochleo-vestibular system through the endolymphatic duct. The luminal fluid of the ES contains a much higher concentration of proteins than any other compartment of the inner ear. This high protein concentration likely contributes to inner ear fluid volume regulation by creating an osmotic gradient between the ES lumen and the interstitial fluid. We characterized the protein profile of the ES luminal fluid of patients (n = 11) with enlarged vestibular aqueducts (EVA) by proteomics. In addition, we investigated differences in the protein profiles between patients with recent hearing deterioration and patients without hearing deterioration. The mean total protein concentration of the luminal fluid was 554.7±94.6 mg/dl. A total of 58 out of 517 spots detected by 2-DE were analyzed by MALDI-TOF MS. The protein profile of the luminal fluid was different from the profile of plasma. Proteins identified from 29 of the spots were also present in the MARC-filtered human plasma; however, the proteins identified from the other 25 spots were not detected in the MARC-filtered human plasma. The most abundant protein in the luminal fluid was albumin-like proteins, but most of them were not detected in MARC-filtered human plasma. The concentration of albumin-like proteins was higher in samples from patients without recent hearing deterioration than in patients with recent hearing deterioration. Consequently, the protein of ES luminal fluid is likely to be originated from both the plasma and the inner ear and considering that inner ear fluid volumes increase abnormally in patients with EVA following recent hearing deterioration, it is tempting to speculate that albumin-like proteins may be involved in the regulation of inner ear fluid volume through creation of an osmotic gradient during pathological conditions such as endolymphatic hydrops.

## Introduction

**Figure 1:**
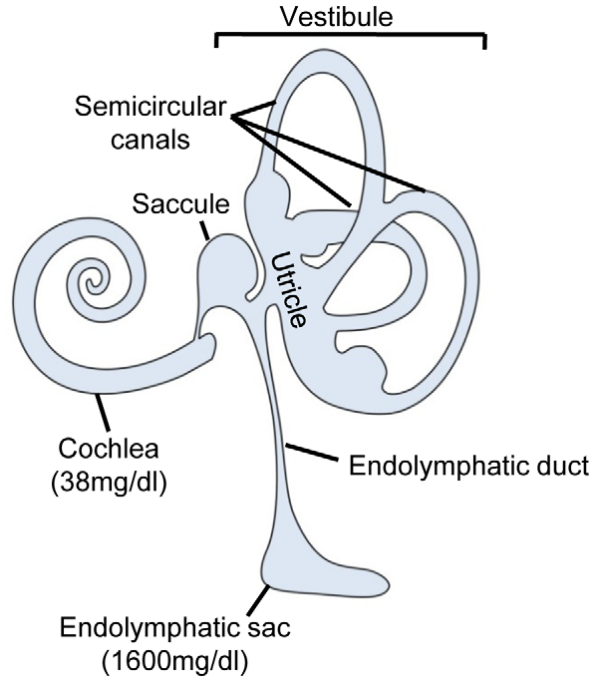
Schematic drawing of the inner ear. The inner ear consists of the cochlea, vestibule and the endolymphatic sac. The cochlea and vestibule, which is composed of the utricle, saccule and three semicircular canals, extend to the endolymphatic sac through the endolymphatic duct. One of the striking compositional differences between cochleo-vestibular endolymph and the luminal fluid of the ES is protein concentration (number in the parenthesis).

The luminal part of the inner ear is filled with a low [Na^+^] and high [K^+^] fluid that is called endolymph [1]. The unique ion composition of this fluid is essential for maintaining hearing and balance by providing K^+^ for mechanotransduction. The endolymphatic sac (ES) is the only non-sensory inner ear organ. The ES is a small structure (∼15 mm^2^) [2] that is an extension of the luminal compartment of the inner ear. It is situated on the posterior fossa dura and is connected to the cochleo-vestibular system through the endolymphatic duct (Fig. 1). The presumed role of the ES is the regulation of the volume of endolymph [3]. If endolymphatic volume regulation is disturbed, serious derangement of inner ear function (i.e., hearing loss and dizziness) may occur. Representative diseases arising from disturbances of endolymphatic volume regulation are Meniere's disease and enlarged vestibular aqueduct (EVA) syndrome. Meniere's disease is a syndrome characterized by symptoms of recurrent vertigo spells, sensorineural hearing loss, tinnitus and aural fullness. EVA syndrome is a congenital disorder that presents profound sensorineural hearing loss. The most common etiology of EVA syndrome in South Asia is the mutation of the SLC26A4 gene [4], which is also known as pendred gene. The primary pathology involved in Meniere's disease is endolymphatic hydrops, which is a phenomenon wherein the volume of endolymph increases abnormally and the endolymphatic space is distended [5]. In addition, most cases of EVA syndrome present with a distended ES, which may reflect increased endolymph volume [6], [7], [8]. So far, the pathophysiological mechanism underlying the increases in endolymphatic volume that occur in these disease states is unclear.

One of the prominent compositional differences between cochleo-vestibular endolymph and the luminal fluid of the ES is protein concentration (Fig. 1). Animal experiments have revealed that the protein concentration of luminal fluid in the ES is extremely high (∼1600 mg/dl), about 40-fold higher than that of cochleo-vestibular endolymph (∼38–60 mg/dl) [9]. The high protein concentration of the luminal fluid of the ES is likely associated with the function of the ES. In addition, the luminal area of the ES is filled with a homogeneous substance that has been characterized as containing heavily glycosylated proteins (proteoglycan) [10], [11]. These proteins have been reported to generate an osmotic gradient that can trigger transport of water into or out of the ES lumen, which consequently alters endolymph volume. However, no reports have identified the components of this homogeneous substance in the luminal fluid of the human ES and no studies have verified whether this homogeneous substance contributes to endolymphatic volume regulation under pathological conditions such as EVA syndrome or Meniere's disease. Currently, little is known about the protein composition of luminal fluid in the human ES, largely because the small volumes of sample available make the analysis of this fluid very difficult. Moreover, the small volume of luminal fluid that can be sampled from the human ES can be easily contaminated by surrounding tissue fluids and blood.

This study examined the luminal fluid from patients with EVA, enabling us to sample a large volume of luminal fluid. We also obtained ES luminal fluid as a control from patients undergoing acoustic tumor by aspirating the fluid after dilution with physiologic saline. The protein profiles of these samples were comprehensively analyzed by proteomics. We compared the protein composition of luminal fluid between patients without sudden hearing deterioration and patients with sudden hearing deterioration where a sudden increase in endolymph volume was suspected. Characterizing the protein composition of luminal fluid and examining the changes in this composition following sudden hearing deterioration will help elucidate the role of the ES in inner ear fluid regulation.

## Results

### Protein concentration of the luminal fluid of the ES from EVA patients

**Figure 2:**
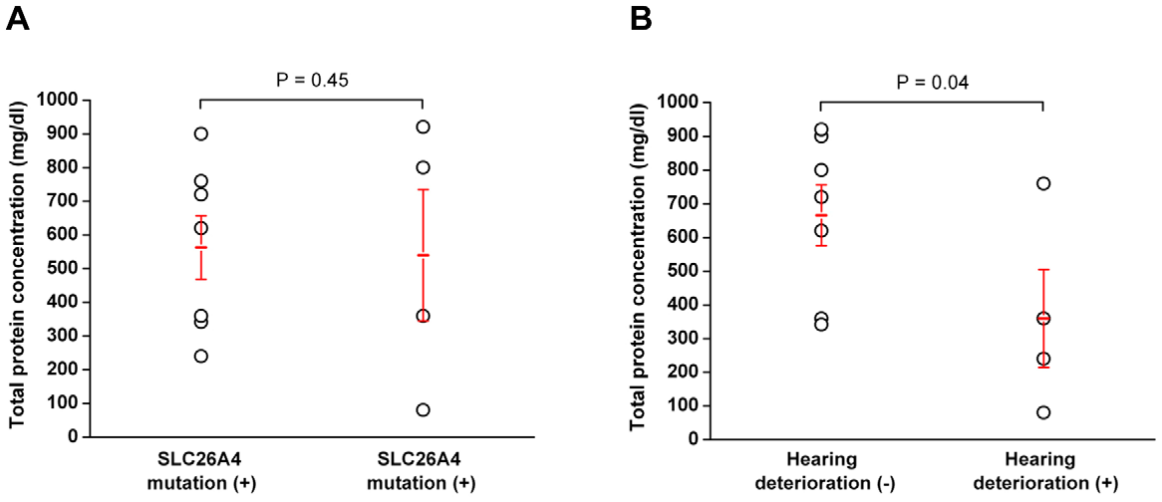
Difference in total protein concentration of the luminal fluid in endolymphatic sac. A: The difference in total protein concentration between patients with the SLC 26A4 mutation versus patients without the SLC26A4 mutation. Mean protein concentrations in patients with the SLC26A4 mutation and in patients without the SLC26A4 mutation were 563.1±94.4 mg/dl and 540.0±194.9 mg/dl, respectively. Total protein concentration was not significantly different (P = 0.45). B: Difference in total protein concentration between patients without recent (<6 months) hearing deterioration [HL (-)] compared to patients with recent hearing deterioration [HL (+)]. Mean protein concentrations in patients without recent hearing loss and with recent hearing loss were 666.0±90.1 mg/dl and 360.0±145.1 mg/dl, respectively. The protein concentration in patients with recent hearing loss was significantly lower than the protein concentration in patients without recent hearing loss (P = 0.04).

The protein concentration of the luminal fluid varied between the samples (80∼920 mg/dl) and the mean total protein concentration was 554.7±94.6 mg/dl (Table 1). We first analyzed the association between the protein concentration and age of patients, which was revealed to have no significance (P>0.05). The mean protein concentration in patients with the SLC26A4 mutation was 563.1±94.4 mg/dl and the mean protein concentration in patients without the SLC26A4 mutation was 540.0±194.9 mg/dl (Fig. 2A). These data indicate that the protein concentration did not associate with the presence of the SLC26A4 mutation (P>0.05). The protein concentration measured for patient 7 (no mutation in SLC26A4 or GJB2) was extremely low relative to the other patients. In contrast, the protein concentration of the other three patients who did not possess the SLC26A4 mutation was not significantly reduced compared to the other patients (P>0.05, Fig. 2A, Table 1). However, the protein concentrations detected in patients with a definitive histories of sudden hearing deterioration within the prior 6 months (recent hearing deterioration) tended to be significantly lower than the protein concentrations detected in patients without a recent hearing deterioration (P = 0.04, Fig. 2B). Specifically, the mean protein concentration in patients without recent hearing loss compared to those with recent hearing deterioration were 666.0±90.1 mg/dl and 360.0±145.1 mg/dl, respectively.

**Table 1:** Demographics and mutation type of patients enrolled in this study.

Patient No.	Age	Sex	Gene (mutation)	Sudden hearing deterioration	Protein concentration (mg/dl)	unnamed protein product (gi|28590)
1	34	M	SLC26A4 (F572 L/H723R)	-	960	61.7
2	20	M	SLC26A4 (H723R/H723R)	-	900	38.2
3	18	F	SLC26A4 (L676Q/IVS7-2A>G)	+	444	4.2
4	3	F	GJB2 (V27I/E114G)	-	660	14.2
5	12	F	SLC26A4(IVS7-2A>G/IVS7-2A>G)	-	620	30.5
6	10	F	SLC26A4 (H723R/IVS7-2A>G)	-	342	61.1
7	16	F	No mutation detected	+	152	0
8	2	M	SLC26A4 (H723R/H723R)	+	612	4.4
9	43	F	No mutation detected	-	920	31.8
10	21	F	SLC26A4 (H723R/H723R)	+	760	49.4
11	8	F	No mutation detected	-	800	40.2

### Difference in protein composition between the luminal fluid of the ES and the plasma in EVA patients

**Figure 3:**
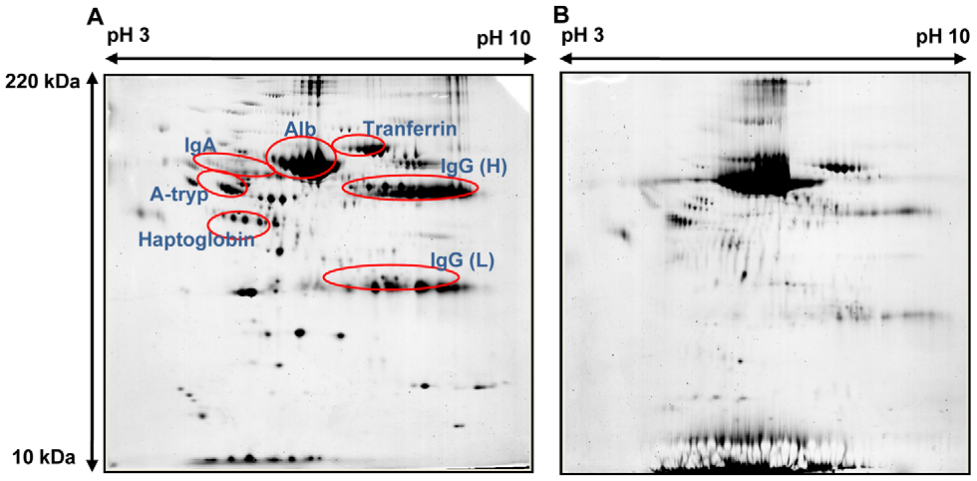
Representative 2-DE of human plasma and luminal fluid from the endolymphatic sac without MARC (patient 1). A: 2-DE of human plasma without MARC. B: 2-DE of luminal fluid from the human endolymphatic sac without MARC. Alb, albumin; IgG (H), IgG heavy chain; IgG (L), IgG light chain; A-tryp, antitrypsin.

The two dimensional gel electrophoresis (2-DE) of the plasma protein composition for patient 1 (Fig. 3A) was similar to the findings in normal human plasma [12]. The six most abundant proteins in the plasma were albumin, IgG (heavy and light chain), IgA, transferrin, antitrypsin, and haptoglobin. However, the protein profile of the luminal fluid from the ES was different from the protein profile of plasma. Albumin, transferrin, IgG heavy chain, IgG light chain, and antitrypsin were also found in the luminal fluid but IgA and haptoglobin were not identified by 2-DE analysis of the luminal fluid (Fig. 3).

**Figure 4:**
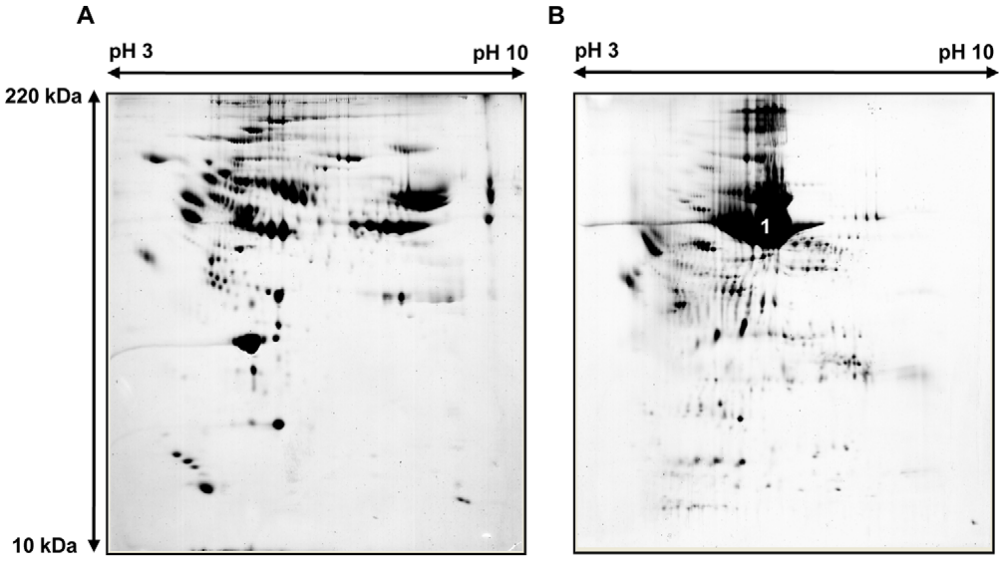
Representative 2-DE of human plasma and luminal fluid from the endolymphatic sac after MARC (patient 1). A: 2-DE of human plasma after MARC. B: 2-DE of luminal fluid from the human endolymphatic sac after MARC.

To further analyze these results, chromatographic separation of the most abundant proteins was performed using multiple affinity removal columns (MARC). The six most abundant plasma proteins in the plasma nearly disappeared after MARC and the 2-DE imaging after MARC was identical to that of normal human plasma (Fig. 4A) [13]. However, the protein composition (according to 2-DE imaging following MARC) was clearly different between plasma and luminal fluid (Fig. 4A and 4B). The most abundant protein spot (spot 1 in Fig. 4B) in the luminal fluid corresponded to that of albumin in plasma, but this spot remained on the gel even after MARC, unlike that of plasma. In addition, there were multiple protein spots that were only seen in the luminal fluid, but not in plasma.

### Identification of proteins in the luminal fluid of ES from EVA patients

**Figure 5:**
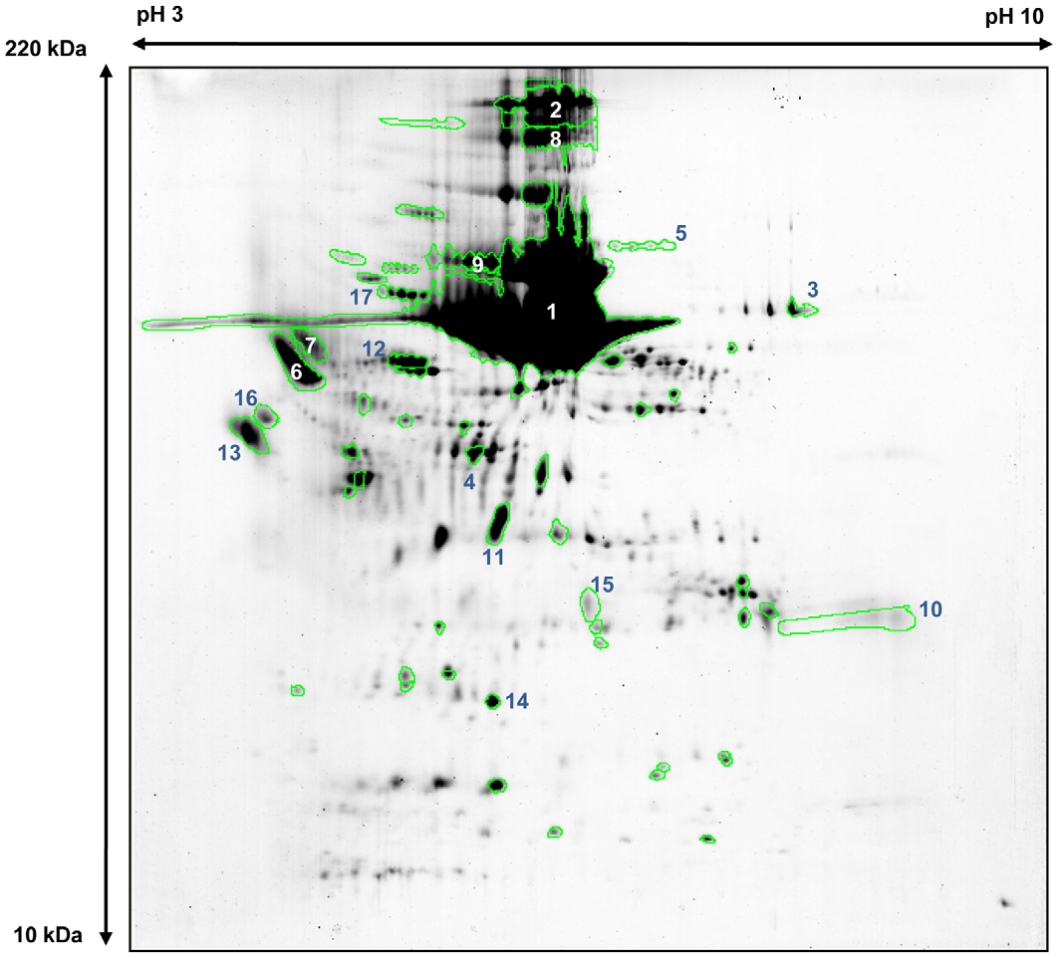
A total of 58 primary spots from the 2-DE analyzed by MALDI/TOF (patient 1). A total of 58 green-bordered spots were analyzed via MALDI/TOF and MS. Among these 58 spots, 17 of the most abundant protein spots were numbered (1∼17) and are described in Tables 3 and 4.

MALDI-TOF MS was performed to identify the proteins represented by each spot in the 2-DE of patient 1. The total number of spots in the 2-DE image from the luminal fluid after MARC was 517. A total of 58 major spots were analyzed via MALDI-TOF MS (Fig. 5). The MALDI-TOF MS analysis identified 55 spots as displaying significant protein scores (p<0.05) and three spots were revealed not to be significant (p>0.05). Twenty-nine of the proteins identified by MALDI-TOF MS were detected in the MARC-filtered human plasma; however, proteins identified from the other twenty-six spots by MALDI-TOF MS were not represented in the MARC-filtered human plasma [13]. Proteins found only in the luminal fluid included hypothetical protein (gi|51476390), hypothetical protein LOC55471 isoform 3 (gi|145701028), unnamed protein product (gi|221043908), minichromosome maintenance complex component 8 isoform 1 (gi|19923727), axonemal beta heavy chain dynein type 11 (gi|15395290), KIAA1505 protein isoform CRA_a (gi|119597447) and Rho GTPase activating protein 23 (gi|224458284), among others. These results are summarized in Figure 5 and Tables 2 and 3. These results suggest that a portion of the proteins in the luminal fluid is originated from the plasma; however, many proteins in the luminal fluid are just as likely to be originated from the ES or from other inner ear compartments.

**Table 2:** Ten most abundant protein spots identified in the luminal fluid of the endolymphatic sac from patient 2 by MALDI-TOF MS.

Spot No.	gi number	Protein Name	Volume (%)
1	gi|51476390	Hypothetical protein*	61.639
2	gi|51476390	Hypothetical protein	5.272
	gi|11493459	PRO 2619	
8	gi|7770217	PRO2675	2.383
6	gi|46981961	growth-inhibiting protein 25	1.858
	gi|28332	unnamed protein product	
	gi|6980544	chain A, alpha1-antichymotrypsin serpin in the delta conformation (partial loop insertion)	
9	gi|228311905	chain A, crystal structure of human serum albumin	0.974
10	gi|145701028	hypothetical protein LOC55471 isoform 3	0.844
11	gi|27065112	chain A, crystal structure of the transthyretin	0.737
12	gi|18655424	chain A, crystallographic analysis of the human vitamin D binding protein	0.618
13	gi|221043908	unnamed protein product	0.484
7	gi|177933	alpha-1-antichymotrypsin precursor	0.332

**Figure 6:**
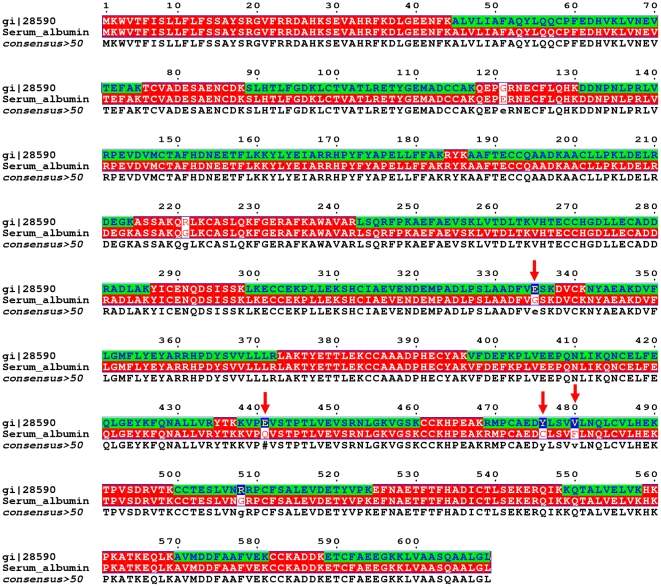
Identification of the most abundant protein spot on the 2-DE by LC-MS/MS (patient 1). Spot 1 on the 2-DE image of Figure 5 and trypsinized protein of patient 1 was analyzed by LC-MS/MS. Proteins were identified from the peptide mass maps using MASCOT to search the protein data bases of Swiss Prot (version 44.1) and GenBank. The protein commonly detected in both samples (part of spot 1 and trypsinized protein of patients 1) was revealed to be unnamed product (gi|28590) by MASCOT (http://www.matrixscience.com/search_form_select.html). It is similar to human albumin (gi|4502027) but has five different amino acids out of 609 total amino acids. Comparison of the amino acid sequences was performed with MultAlin (http://multalin.toulouse.inra.fr/multalin/multalin.html). The amino acid sequence shown in the first row is the unnamed protein product (gi|28590), the second row is human serum albumin (gi|4502027) and the third row is the consensus between the two. Identified sequences are marked with green rectangle and blue ones are the unique sequence of the unnamed protein product (gi|28590). Arrows indicates the peptides for the unnamed protein product (gi|28590) of which the ion scores more than 42 (P<0.05).

*, hypothetical protein (gi|51476390) was finally revealed to be a mixture of albumin-like proteins by LC-MS/MS (See table 4, figure 6, and figure S1).

**Table 3:** Ten most abundant protein spots that were not found in human plasma but were found in the luminal fluid of the endolymphatic sac from patient 2 by MALDI-TOF MS.

Spot No.	gi number	Protein Name	Volume (%)
1	gi|51476390	Hypothetical protein*	61.639
2	gi|51476390	Hypothetical protein	5.272
	gi|11493459	PRO 2619	
10	gi|145701028	hypothetical protein LOC55471 isoform 3	0.844
13	gi|221043908	unnamed protein product	0.484
14	gi|19923727	minichromosome maintenance complex component 8 isoform 1	0.204
15	gi|15395290	axonemal beta heavy chain dynein type 11	0.2
16	gi|119597447	KIAA1505 protein, isoform CRA_a	0.197
4	gi|51476390	hypothetical protein	0.197
5	gi|51476390	hypothetical protein	0.162
17	gi|224458284	Rho GTPase activating protein 23	0.12

*, hypothetical protein (gi|51476390) was finally revealed to be a mixture of albumin-like proteins by LC-MS/MS (See table 4, figure 6, and figure S1).

We performed LC-MS/MS with part of the most abundant spot (spot 1 in Fig. 5) in the 2-DE image, which was identified as a hypothetical protein (gi|51416390) by MALDI-TOF MS, and also the trypsinized protein of patient 1 to confirm the result. Finally, the protein commonly detected in both samples (part of spot 1 and trypsinized protein of patients 1) was revealed to be an unnamed product (gi|28590), which was also similar to human albumin (gi|4502027) but has a total of 6 different amino acids out of 609 amino acids (Fig. 6, Table 4, and Fig. S1); sequence coverage on LC-MS/MS was 66%, score was 1659 and four unique peptide sequence for the unnamed protein product (gi|28590) was detected (ion scores >42 and p<0.05). However, several albumin-like proteins were also detected in LC-MS/MS in each sample such as chain A, crystal structure of human serum albumin (gi|3212456; Mr, 66472; pI value, 5.67; score, 1657; sequence coverage 70%), unnamed protein product (gi|194391080; Mr, 70362; pI value, 5.77; score, 763; sequence coverage 32%), unnamed protein product (gi|194391112; Mr, 59573; pI value, 6.88; score, 652; sequence coverage 23%), PRO2675 (gi|7770217; Mr, 32574; pI value, 6.14; score, 504; sequence coverage 33%) and albumin, isoform CRA_t (gi|119626083; Mr, 58652; pI value, 6.66; score, 356; sequence coverage 27%) (Table S1). Thus, it is more likely that spot 1 is a mixture of albumin-like proteins than just consisting of one protein.

**Table 4:** Unique peptide sequences for the albumin-like unnamed protein product (gi|28590) identified by LC-MS/MS.

Sequence (start-end)	Peptide sequence	Ion score
311–337	SHCIAEVENDEMPADLPSLAADFVESK	45
438–452	KVPEVSTPTLVEVSR	57
470–490	RMPCAEDYLSVVLNQLCVLHEK	57

Nominal mass of the unnamed protein product (gi|28590) is 69295, calculated pI value in Mascot search was 5.92, and sequence coverage was 66%. MS/MS spectra of above peptides are provided in figure S1.

### Recent hearing deterioration in EVA patients correlates with decreased levels of albumin-like proteins

**Figure 7:**
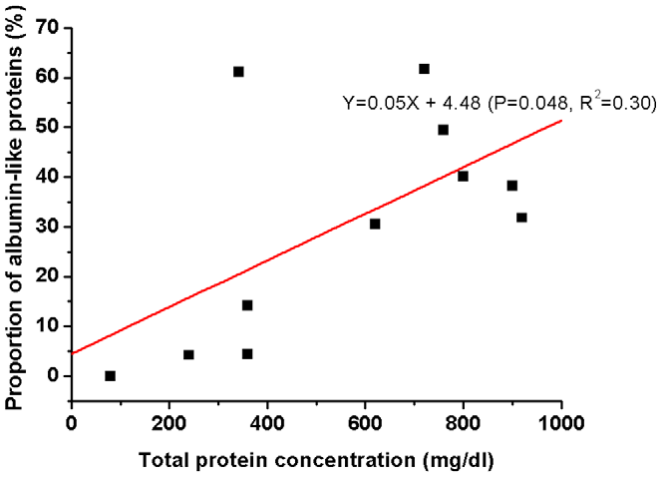
The relationship of albumin-like proteins and total protein concentration in the luminal fluid of the endolymphatic sac. The proportion of total protein tended to be more represented by albumin-like proteins as the protein concentration of the luminal fluid increased. (Y = 0.05X + 4.48, P = 0.048, R^2^ = 0.30).

**Figure 8:**
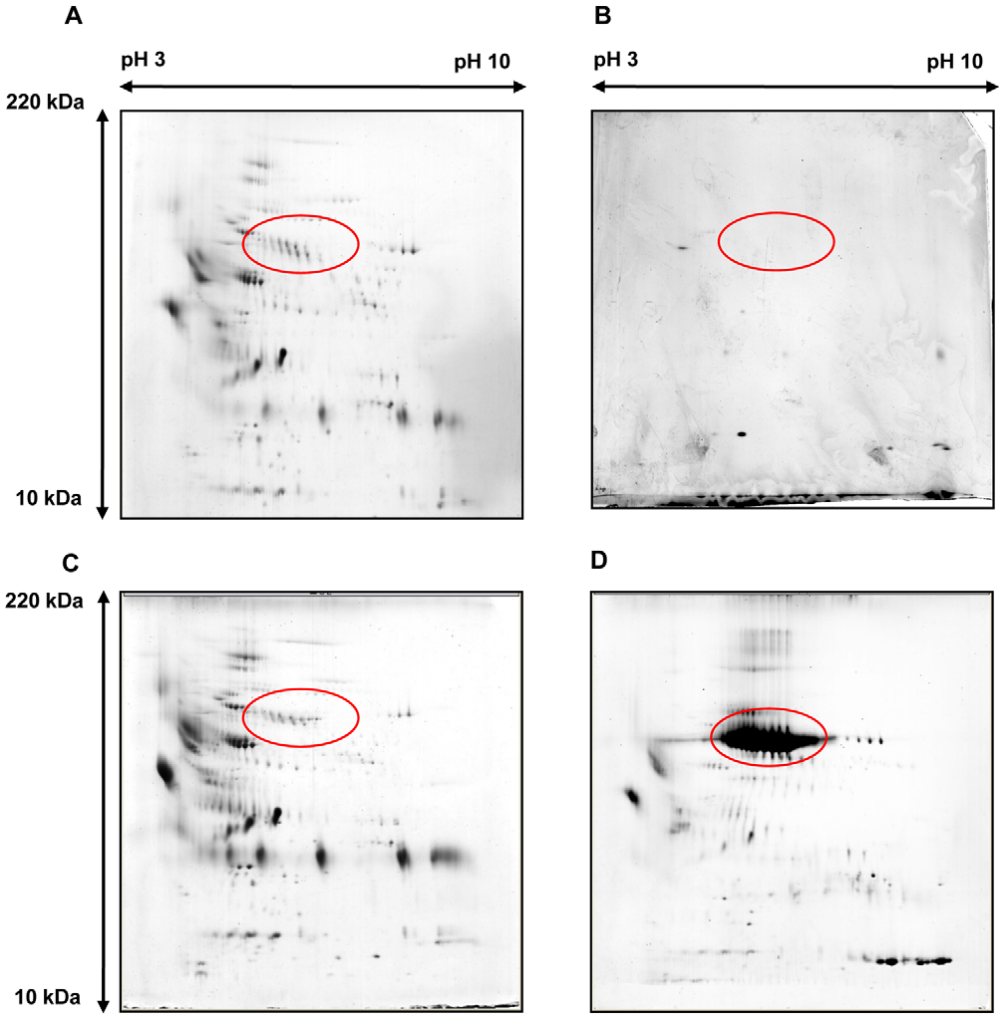
2-DE image of patients with recent (<6 months) hearing deterioration. The proportion of albumin-like proteins in patients with recent hearing deterioration decreased. Red circles in the figure represent albumin-like proteins. A: 2DE image of patient 3; B: 2DE image of patient 7; C: 2DE image of patient 8; D: 2DE image of patient 10.

**Figure 9:**
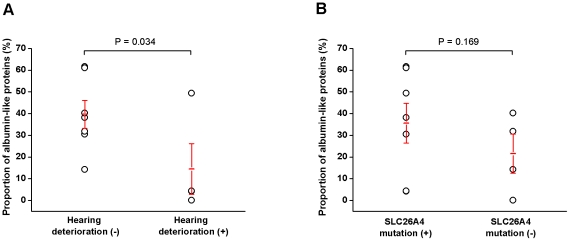
Differences in the proportions of albumin-like proteins. A: The proportion of albumin-like proteins in patients without recent (<6 months) hearing deterioration was 39.7±6.4%, whereas the proportion of albumin-like proteins in patients with recent hearing deterioration was 14.5±11.7%. The proportion of albumin-like proteins was decreased in patients with recent hearing deterioration (P = 0.034). B: The proportion of albumin-like proteins in patients with the SLC26A4 mutation was 35.6 ±39.7±6.4%, whereas the proportion of albumin-like proteins in patients without the SLC26A mutation was 21.6±9.0%. The proportion of albumin-like proteins did not differ significantly according to mutation status.

This study analyzed the difference in the proportion of the largest spot (albumin-like proteins, e.g., spot 1 in Fig. 5) in the 2-DE images of each patient. The proportion of total protein tended to be more represented by albumin-like proteins as the protein concentration of the luminal fluid increased (p = 0.048, R^2^ = 0.30, Fig. 7). In comparing the protein compositions of the luminal fluid between patients who suffered recent (<6 months) hearing deterioration versus patients who did not, the most striking difference observed was that patients with a history of recent hearing deterioration showed a decreased proportion of the albumin-like proteins in the 2-DE image (Fig. 8). The proportion of albumin-like proteins in patients without recent hearing deterioration was 39.7±6.4%, whereas the proportion of albumin-like proteins in patients with recent hearing deterioration was 14.5±11.7% (Fig. 9A). The proportion of albumin-like proteins was significantly decreased in patients who presented with recent hearing deterioration (p = 0.034, Fig. 9A). The proportion of the albumin-like proteins did not differ significantly with respect to the presence of the SLC26A4 mutation. Most patients were determined to have the SLC26A4 mutation, although one patient presented the GJB2 mutation (patient 4) and several other patients (patient 7, 8, and 11) did not possess either of the two mutations. The proportion of albumin-like proteins did not differ between the patients with the SLC26A4 mutation and the patients without the mutation (P>0.05, Fig. 9B). The proportion of albumin-like proteins was very low in patient 4 (GJB2 mutation) and patient 7 (no mutation) (Table 1); however, the number of patients with this mutation was too small to reveal any relationship between the mutation and the proportion of albumin-like proteins.

### Albumin-like proteins in the luminal fluid from the ES of acoustic schwannoma as a control for no hydrops

**Figure 10:**
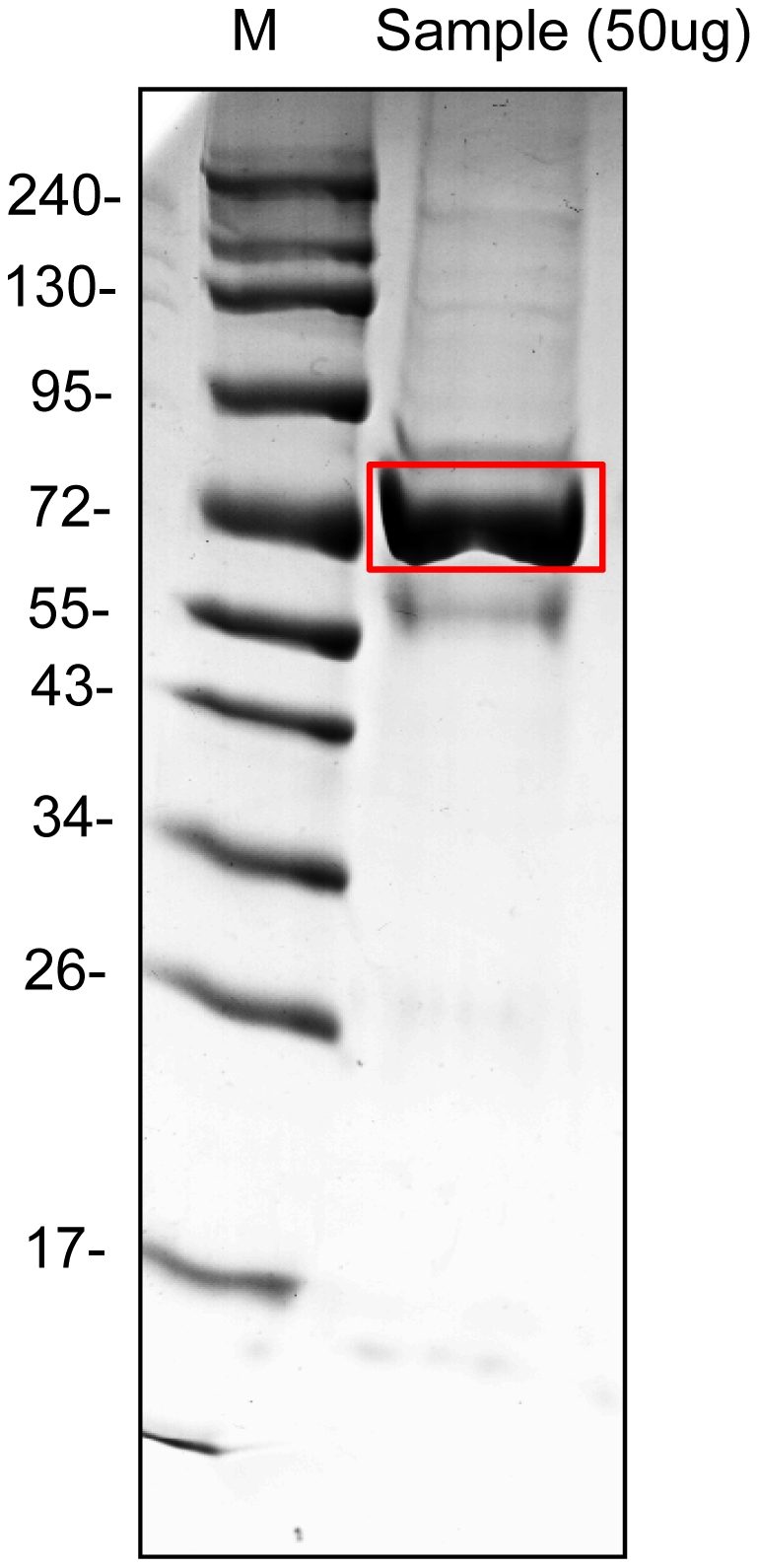
1-DE of the luminal fluid from the endolymphatic sac of controls. The luminal fluid from the endolymphatic sac of controls was obtained during acoustic tumor surgery via a translabyrinthine approach. The highest signal band was excised (red line) and liquid chromatography mass spectrometry was performed. The molecular weight of the band corresponded to the albumin-like proteins from the 2-DE analysis.

This study sought to determine whether the albumin-like proteins were also expressed under normal conditions by identifying proteins in the luminal fluid from the ES of acoustic schwannoma as a control for no hydrops. The highest signal band detected by 1-DE gel analysis of samples from these patients corresponded to the molecular weight of the albumin-like proteins in the 2-DE gel image of the luminal fluid from EVA patients (Fig. 10). The band was excised and LC-MS/MS was performed. Four types of proteins were identified by this analysis: albumin or albumin-like protein (Table 5), immunoglobulins, ribosomal proteins, and interferon regulatory factor 7. Albumin-like proteins were also present in the luminal fluid of controls, and two of them, an unnamed protein product (gi|28590) and a chain A, crystal structure of human serum albumin, were identified by the LC-MS/MS analysis with ES luminal fluid of EVA patients (Table 5 and Table S2). The albumin-like proteins are likely to normally exist in the luminal fluid of ES and its concentration is likely to change in some pathologic conditions such as sudden hearing deterioration in EVA patients.

**Table 5:** Albumin or albumin-like protein identified by LC-MS/MS in the luminal fluid from a normal endolymphatic sac.

gi number	protein name	M_r_	pI	C (%)
gi|3212456	Chain A, Crystal Structure Of Human Serum Albumin	66472	5.67	52
gi|4502027	Serum albumin preproprotein [Homo sapiens]	69366	5.92	50
gi|78101694	Chain A, Human Serum Albumin Complexed With Myristate And Azapropazone	66444	5.57	52
gi|28590	unnamed protein product [Homo sapiens]	69295	5.92	50

M_r_, nominal mass; pI, calculated pI value in Mascot search; C, sequence coverage.

## Discussion

The role of the ES is unclear but it has been suggested to be involved in inner ear homeostasis and endolymphatic volume regulation. Under normal conditions, endolymph flow from the cochleovestibular system to the ES is thought to be minimal. However, under pathological conditions (e.g., during endolymphatic hydrops), endolymph flows from the cochleovestibular system to the ES [3]. In addition, the ES regulates endolymph volume by absorbing fluid from the interstitial space [14]. This volume flow is thought to serve as an important regulatory mechanism contributing to the control of normal fluid volume. One of the suggested mechanisms involved in endolymphatic volume regulation is the transport of water into or out of the ES lumen, which depends on the magnitude of the osmotic gradient between the lumen and the interstitial space. Rask-Anderson et al., proposed that the intraluminal homogeneous substance in the ES creates an osmotic gradient that contributes to endolymphatic volume regulation, based on animal experiments [14]. The amount of this homogeneous substance decreased in response to increased inner ear volume load, which decreased the osmotic gradient between the endolymph and the interstitial space to enhance the movement of endolymph into the interstitial space. In contrast, levels of the homogenous substance increased in response to inner ear volume depletion, which enhanced the movement of fluid from the interstitial space to the lumen of the ES. This response occurs within a matter of minutes and has been observed after systemic treatment with glycerol in several animal experiments [15], [16]. The homogeneous substance is believed to be originated from the light cells, a type of ES epithelial cell and is degraded by macrophages. The homogeneous substance is believed to contain macromolecular complexes that consist of proteoglycans [10], [11] or glycoproteins [17], [18], which have been detected by autoradiographic methods through the uptake of radioactive sulfur [10]. However, no comprehensive study has been performed to identify the components of this substance. According to our results, we believe that the homogeneous substance is primarily composed of the albumin-like proteins. Albumin undergoes glycosylation [19] and contains disulphide bonds [20], characteristics similar to the homogeneous substance described above. In EVA patients, head trauma or noise exposure can induce labyrinthine hemorrhage, which consequently increases the protein concentration and volume of the endolymph [8], [21]. Subsequently, a sudden loss of hearing is frequently manifested [22]. In our series of experiments, most patients who displayed a definite history of sudden hearing deterioration within the prior 6 months showed a decreased proportion of the albumin-like proteins in their luminal ES fluid. In contrast, patients without recent hearing deterioration showed a higher proportion of albumin-like proteins. We speculate that levels of albumin-like proteins decreased in response to inner ear fluid volume or protein increases to regulate inner ear fluid volume by decreasing the magnitude of the osmotic gradient. In this study, the maximum difference of osmotic pressure induced by albumin concentration change is 0.88mOsm/kg; the osmotic pressure induced by albumin can be roughly calculated in patient 1, in whom the volume of luminal fluid was stable, as 0.88 mOsm/kg [(960mg/dl ×0.617)/67,000 (molecular weight of albumin)], and as nearly 0 mOsm/kg in patient 7, in whom hearing deterioration had recently occurred. Although other factors may contribute to the osmotic gradient between the luminal fluid and interstitial fluid, it seems that the osmotic pressure change induced by the albumin-like proteins plays an important role in the exchange of fluid between the two compartments, as does occur in other parts of the human body. At first, the most abundant protein spot (spot 1 in Fig. 5) was identified as hypothetical protein (gi|51476390) by MALDI-TOF MS, which is similar to human serum albumin. The protein spot area represented 61.6% of total spots in the 2-DE imaging. In addition to spot 1, four other spots (spots 2, 3, and 4, Fig. 5) were revealed as the same protein. However, it is reasonable that one spot on 2-DE image contains more than one protein, so we performed LC-MS/MS to confirm the protein component of spot 1, which was finally identified as a mixture of albumin-like proteins. The LC-MS/MS result is likely to be more reliable since Peptide Mass Fingerprinting (PMF)-based protein identification can run into difficulties with protein mixtures, low abundant amounts of protein, and post-translational modifications. Thus, it has been found that MS/MS-based protein identification is more accurate than PMF-based identification [23]. We also identified four albumin-like proteins in controls, and two of them exactly coincided with the unnamed protein product (gi|28590) and the chain A, crystal structure of human serum albumin (gi|3212456) in the luminal fluid of EVA patients, respectively. Consequently, albumin-like proteins are thought to normally exist in the luminal fluid ES and play an important role in regulating inner ear fluid volume in pathologic conditions by creating an osmotic gradient between the luminal and interstitial spaces.

Protein analysis of the luminal fluid of guinea pig ES has been previously reported [9]. In that study, protein profiles of the luminal fluid via 2-DE showed that the major plasma proteins were also found in the luminal fluid. This result was similar to the results of our study; however, we found that the protein profile was very different between the luminal fluid and plasma following MARC. In addition, the previous study did not perform MS to identify these proteins. The most abundant protein in the luminal fluid was found to be albumin-like proteins, but most of them differ from the form of human plasma albumin. These proteins did not disappear even after MARC, which is different from the case of plasma. There are several possibilities as to why this protein was not filtered. First, it is possible that the amount of albumin-like proteins was too high and could not be filtered completely. However, it does not seem probable because the protein and albumin concentration of the ES luminal fluid is not higher than plasma. Second, the conventional MARC we used was for human plasma samples and so the ES luminal fluid might not have been filtered effectively. Third, the epitope of the albumin-like proteins in the ES luminal fluid might have been different from that of plasma albumin, resulting in the ineffective filtering of the albumin-like proteins by MARC. If the third possibility is correct, it suggests that the ES luminal fluid albumin-like proteins and plasma albumin are likely to be different. Besides albumin-like proteins such as the unnamed protein product (gi|28590), unnamed protein product (gi|194391080) and unnamed protein product (gi|194391112), several other proteins that were not found in the plasma were found in the luminal fluid. These proteins included: hypothetical protein LOC5547 isoform 3 (gi|145701028), which is identical to protein midA homolog mitochondrial isoform 3, a kind of mitochondrial protein (http://www.ncbi.nlm.nih.gov/protein/145701028); unnamed protein product (gi|221043908), which is a protein similar to the ligand binding domain of the retinoid X receptor (http://www.ncbi.nlm.nih.gov/nuccore/221043907); minichromosome maintenance complex component 8 isoform 1 (gi|19923727), which is involved in cell cycle transcription (http://www.ncbi.nlm.nih.gov/sites/entrez?db=gene&cmd=retrieve&list_uids=84515); axonemal beta heavy chain dynein type 11 (gi|15395290), which is a microtubule-tubule dependent motor ATPase that is involved in the movement of cilia (http://www.ncbi.nlm.nih.gov/sites/entrez?db=gene&cmd=retrieve&list_uids=8701); KIAA1505 protein, isoform CRA_a (gi|119597447), a protein coded from CCDC146 (coiled coil domain containing 146) with an unknown function (http://www.ncbi.nlm.nih.gov/protein/119597447); and Rho GTPase activating protein 23 (gi|224458284), which is involved in signal transduction through transmembrane receptors (http://www.ncbi.nlm.nih.gov/protein/224458284). The ES has been shown to contain cellular components originated from inner ear compartments. Considerable evidence has been reported to indicate that damaged inner ear tissues and cells (including degraded otoconia-like or stereocilia-like structures) are found in the human ES [24]. These tissues and cells are thought to be removed by macrophages and epithelial cells of the ES through phagocytic activity [15], [25]. Therefore, the proteins listed above are likely derived from cellular components of the luminal fluid of the ES or involved in signal transduction by binding to specific molecules of the inner ear. Our results indicate that the luminal fluid of the ES not only contains several identical proteins to plasma but also contains proteins that were not identified in plasma. This implies that the luminal fluid of the ES is originated from both plasma and the inner ear. To clarify the origin of luminal fluid, protein profiles of human perilymph and CSF should be comprehensively analyzed. However, obtaining sufficient perilymph and CSF for protein analysis from EVA patients may give rise to ethical problems. Additionally, perilymph samples can be easily contaminated by surrounding tissue fluids and CSF through the cochlea aqueduct.

This is the first report that has analyzed and identified the protein profile of luminal fluid from the human ES. It is tempting to speculate that the proteins in the ES luminal fluid are originated from both the plasma and the inner ear. Among the proteins in the luminal fluid, presumably, the albumin-like proteins contributes to the regulation of inner ear fluid volume by creating an osmotic gradient between the luminal fluid of the ES lumen and the surrounding interstitial space, which may be especially important during pathological conditions such as endolymphatic hydrops.

## Materials and Methods

### Subjects

This study was approved by the institutional review board of the Yonsei University College of Medicine and informed written consent was obtained from each of the participants. Among the patients with profound hearing loss who visited Severance Hospital for cochlear implantation from January, 2009 to July, 2010, a total of 11 sequential patients with bilateral EVA (as confirmed by TBCT and MRI) as a disease group and two patients with acoustic schwannoma as a control group were enrolled in the study. The patients with EVA displayed hearing thresholds greater than 100 dB on pure tone audiogram or auditory brainstem response. Vestibular function was preserved according to the caloric test in six patients after surgery. All patients underwent molecular testing for the SLC26A4 and GJB2 mutations. Seven patients were found to have homogenous or compound heterogeneous mutations of SLC26A4. One patient was found to have compound heterogeneous GJB2 mutations and three patients did not showed any mutations upon molecular testing (Table 1). Four patients (Patient 3, 7, 8 and 10) had a definitive history or audiogram of sudden hearing loss within 6 months prior to cochlear implantation. They did not undergo any medical treatment for sudden hearing loss, and their hearing did not improve before surgery. Seven patients did not display sudden hearing loss within 2 years prior to cochlear implantation.

### Sampling of blood and ES luminal fluid

**Figure 11:**
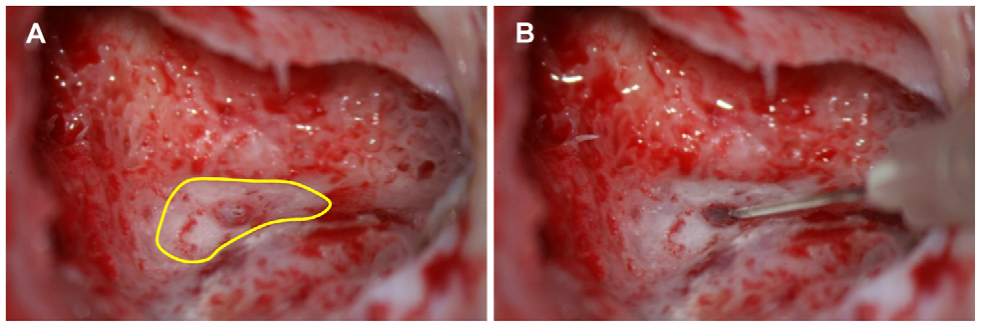
Sampling of the luminal fluid from the endolymphatic sac of EVA patients. The endolymphatic sac was exposed minimally by the thinning and peeling off of the bony covering (A) and the sac was punctured with a 30-gauge needle syringe. The luminal fluid was then gently aspirated (B).

During cochlear implantation, the ES was minimally exposed by the thinning and peeling off of the bony covering (Fig. 11A). Bleeding from the tissue was carefully controlled with a 0.001% epinephrine soaked cotton ball and dried to avoid contamination from blood and tissue fluids. The sac was punctured with a 30-gauge needle syringe and luminal fluid was gently aspirated (200∼300 µl) (Fig. 11B). The luminal fluid from the ES of the controls was obtained during acoustic tumor surgery via a translabyrinthine approach. Because the amount of luminal fluid in normal ES is too small (<4 µl) [26], direct aspiration without contamination from normal ES was very difficult. Therefore, we infused 200 µl of normal saline into the ES and then immediately aspirated approximately the same volume of injected fluid. All fluid samples were immediately stored at −80°C until analysis. Peripheral blood was sampled in an ethylenediaminetetraacetic acid containing tube for 2-DE analysis from one of the patients (Table 1, patient 1). Plasma was separated from the blood immediately and was stored at −80°C until analysis.

### Preparation of samples for 2-DE

Whole luminal fluid (150 µl) from the ES or plasma of EVA patients was desalted by trichloroacetic acid (TCA)/acetone precipitation. First, 50% (w/v) TCA (Sigma, St. Louis, MO) was added to reach a final TCA concentration of 5–8%. The sample was centrifuged at 14,000 g for 15 min and the supernatant was discarded. Then 200 µl of cold acetone was added. After incubation for 15 min on ice, the sample was centrifuged at 14,000 g for 15 min and the acetone was discarded. The remaining pellet was dried and dissolved in the sample buffer for 2 DE. We used multiple affinity removal columns (MARC, Agilent, Palo Alto, CA) to deplete the most abundant proteins (i.e., albumin, transferrin, IgG, IgA, haptoglobin, and anti-trypsin) in the endolymph and blood samples. However, this procedure was not used for the sample from patient 8, as the protein concentration was too low (total 152 µg of protein separated). A 4.6×100 mm MARC with a binding capacity for 20 µl of human plasma was used for MARC. Chromatographic separation of the abundant proteins by MARC was carried out using a mobile phase reagent kit according to a standard LC protocol provided by the manufacturer. Crude samples were diluted five times with Buffer A containing protease inhibitors (COM-PLETE^TM^, Roche, Branchburg, NJ) and filtered through 0.22 µm spin filters by spinning at 16,000 g at room temperature for 1–2 min. The sample was injected and flow-through fractions were collected and stored at −20°C until use. To resolve depleted proteins on 2-D gels, flow-through fractions from MARC were pooled and precipitated with a pre-cooled solution of 10% TCA for 1 h at −20°C. After washing with ice-cold acetone, the pellets were resolublized in the 2-DE sample buffer. The protein concentration was quantified by the Bradford protein assay following MARC.

### 2-DE

2-DE was carried out essentially as described [13]. Aliquots in sample buffer (7 M urea, 2 M thiourea, 4.5% CHAPS, 10 mM DTE and 40 mM Tris, pH 8.8) were applied to immobilized pH 3–10 nonlinear gradient strips (Amersham Biosciences, Uppsala, Sweden). Isoelectric focusing was performed at 80,000 Vh. The second dimension was analyzed on 9–16% linear gradient polyacrylamide gels (18 cm ×20 cm ×1.5 cm) at a constant 40 mA per gel for approximately 5 hours. After protein fixation in 40% methanol and 5% phosphoric acid for 1 hr, the gels were stained with Coomassie Brilliant Blue (CBB) G-250 for 12 hours (except for patient 7 where the protein concentration was 152 µg and silver staining was performed). The gels were destained with H_2_O, scanned in a Bio-Rad GS710 densitometer and converted into electronic files.

### Identification of protein by matrix-assisted laser desorption/ionization - time of flight mass spectrometer (MALDI-TOF MS)

For 2-D gel mapping of the luminal fluid proteome, 58 major proteins were identified by mass finger printing or by matching with various internal 2-DE maps. Protein spots excised from 2-DE gels were destained, reduced, alkylated and digested with trypsin (Promega, Madison, WI) as previously described [27]. For MALDI-TOF MS analysis, the peptides were concentrated by a POROS R2, Oligo R3 column (Applied Biosystems, Fostercity, CA, USA). After washing the column with 70% acetonitrile, 100% acetonitrile and then 50 mM ammonium bicarbonate, samples were applied to the R2, R3 column and eluted with cyano-4-hydroxycinamic acid (CHCA) (Sigma, St. Louis, MO) dissolved in 70% acetonitrile and 2% formic acid onto the MALDI plate (Opti-TOF™ 384-well Insert, Applied Biosystems). MALDI-TOF MS was performed on 4800 MALDI-TOF/TOF™ Analyzer (Applied Biosystems) equipped with a 355-nm Nd:YAG laser. The pressure in the TOF analyzer is approximately 7.6e-07 Torr. The mass spectra were obtained in the reflectron mode with an accelerating voltage of 20 kV and sum from either 500 laser pulses and calibrated using the 4700 calibration mixture (Applied Biosystems). Proteins were identified from the peptide mass maps using MASCOT (http://www.matrixscience.com/search_form_select.html), which searched the protein databases of the NCBI non-redundant human database containing 115818 entries(downloaded on 05/09/2009). To confirm the results of MALDI-TOF MS, especially spot 1 which occupied the largest part in 2-DE image (Fig. 5), liquid column mass spectrometry (LC-MS/MS) with the part of spot 1 in 2-DE of patient 1 was performed as described below. This result was confirmed by comparing the result of LC-MS/MS with the trypsinized protein from patient 1(Fig. 5).

### 1-DE

The volume of luminal fluid obtained from normal ES was too small to perform 2-DE analysis. Therefore, we performed 1-DE to examine whether the major proteins in the luminal fluid of the normal ES were the same as those in the luminal fluid of EVA patients. Because the protein concentration of the diluted luminal fluid aspirated during surgery was too small for protein analysis, two samples from two normal ES were loaded together. The total amount of protein in the diluted luminal fluid was 25.3 µg for one sample. We did not use MARC for these samples due to the small amount of total protein. The samples were lyophilized and dissolved in 15 µl of distilled water. Samples were subjected to SDS gel electrophoresis on a 12% polyacrylamide gel and stained with CBB. The gel band for the most prominent protein was excised. After reduction with DTT and alkylation with iodoacetamide, the band was treated with trypsin to digest the proteins *in situ*. Bands were washed with 10 mM ammonium bicarbonate and 50% ACN, swollen in digestion buffer containing 50 mM ammonium bicarbonate, 5 mM CaCl_2_, and 1 µg of trypsin, which were then incubated at 37°C for 12 hours. Peptides were recovered by two cycles of extraction with 50 mM ammonium bicarbonate and 100% ACN. The resulting peptide extracts were pooled, lyophilized and stored at −20°C. To identify the protein profile of 1-DE bands, LC-MS/MS was performed.

### Identification of proteins by liquid column mass spectrometry (LC-MS/MS)

Nano LC–MS/MS analysis was performed on an agilent 1100 Series nano-LC and LTQ- mass spectrometer (Thermo Electron, San Jose, CA). The capillary column used for LC–MS/MS analysis (150 mm ×0.075 mm) was obtained from Proxeon (Odense M, Denmark) and slurry packed in house with 5 µm, 100 Å pore size Magic C18 stationary phase (Michrom Bioresources, Auburn, CA). The mobile phase A for the LC separation was 0.1% formic acid in deionized water and the mobile phase B was 0.1% formic acid in acetonitrile. The chromatography gradient was set up to give a linear increase from 5% B to 35% B in 100 min and from 40% B to 60% B in 10 min and from 60% B to 80% B in 20 min. The flow rate was maintained at 300 nL/min after splitting. Mass spectra were acquired using data-dependent acquisition with full mass scan (400–1800 m/z) followed by MS/MS scans. Each MS/MS scan acquired was an average of one microscans on the LTQ. The temperature of the ion transfer tube was controlled at 200°C and the spray was 1.5.0–2.0 kV. The normalized collision energy was set at 35% for MS/MS. Mass tolerances of 1.2 Da and 0.6 Da were used for precursor and fragment ions, respectively. Peptides were allowed to be variably oxidized at methionine residues and to be variably carboxymethylated at cystein.

### Data analysis

Detection and determination of volume (%) for each spot was performed with Image Master Platinum 5 (GE Healthcare, Piscataway, NJ). Data are presented as the mean values ± SE. Significance between two groups was calculated with an unpaired t-test and the relationship between the total protein concentration and the proportion of the unnamed protein product (gi|28590) was analyzed with linear regression. Differences and linear relationships were considered significant at p<0.05.

## Supporting Information

Figure S1 **MS/MS spectra of unique peptide sequences for unnamed protein product (gi|28590) revealed by LC-MS/MS.** A: MS/MS spectrum of amino acid sequence from 311-337 (peptide sequence: SHCIAEVENDEMPADLPSLAADFVESK, ion socre:45). B: MS/MS spectrum of amino acid sequence from 438-452 (peptide sequence: KVPEVSTPTLVEVSR, ion score:57). C: MS/MS spectrum of amino acid sequence from 470-490 (peptide sequence: RMPCAEDYLSVVLNQLCVLHEK, ion score: 57).
(TIF)

Table S1 **Albumin-like proteins identified by LC-MS/MS except unnamed protein product (gi|28590) in each sample from part of spot 1 (** **Fig. 5** **) and trypsinized protein of patients 1.**
(XLS)

Table S2 **Albumin or albumin-like proteins identified by LC-MS/MS in the luminal fluid from a normal endolymphatic sac.**
(XLS)
