# Supplementary figures and images for: Albumin-Like Protein is the Major Protein Constituent of Luminal Fluid in the Human Endolymphatic Sac
Source: PLoS One. 2011 Jun 29;6(6):e21656. doi: 10.1371/journal.pone.0021656 (PMC3126852; doi:10.1371/journal.pone.0021656)

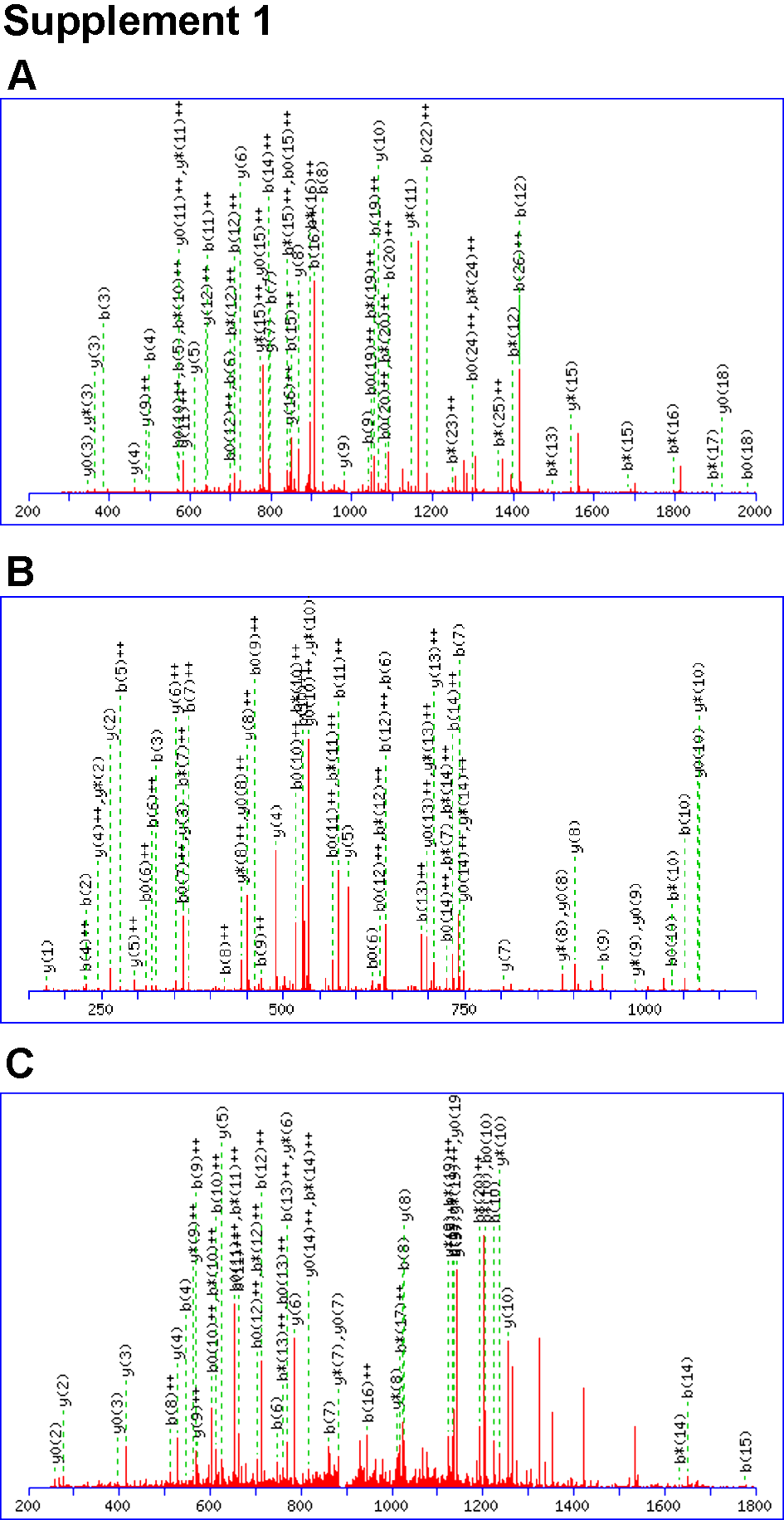

Supplement: Figure S1 — MS/MS spectra of unique peptide sequences for unnamed protein product (gi|28590) revealed by LC-MS/MS. A: MS/MS spectrum of amino acid sequence from 311-337 (peptide sequence: SHCIAEVENDEMPADLPSLAADFVESK, ion socre:45). B: MS/MS spectrum of amino acid sequence from 438-452 (peptide sequence: KVPEVSTPTLVEVSR, ion score:57). C: MS/MS spectrum of amino acid sequence from 470-490 (peptide sequence: RMPCAEDYLSVVLNQLCVLHEK, ion score: 57). (TIF) [file pone.0021656.s001.tif]
